# High-fidelity diabetic retina fundus image synthesis from freestyle lesion maps

**DOI:** 10.1364/BOE.477906

**Published:** 2023-01-04

**Authors:** Benjamin Hou

**Affiliations:** Biomedical Image Analysis, Imperial College London, UK

## Abstract

Retina fundus imaging for diagnosing diabetic retinopathy (DR) is an efficient and patient-friendly modality, where many high-resolution images can be easily obtained for accurate diagnosis. With the advancements of deep learning, data-driven models may facilitate the process of high-throughput diagnosis especially in areas with less availability of certified human experts. Many datasets of DR already exist for training learning-based models. However, most are often unbalanced, do not have a large enough sample count, or both. This paper proposes a two-stage pipeline for generating photo-realistic retinal fundus images based on either artificially generated or free-hand drawn semantic lesion maps. The first stage uses a conditional StyleGAN to generate synthetic lesion maps based on a DR severity grade. The second stage then uses GauGAN to convert the synthetic lesion maps into high resolution fundus images. We evaluate the photo-realism of generated images using the Fréchet inception distance (FID), and show the efficacy of our pipeline through downstream tasks, such as; dataset augmentation for automatic DR grading and lesion segmentation.

## 1. Introduction

Diabetic Retinopathy (DR) is the fastest growing cause of blindness, with approximately 537 million diabetic patients (aged between 20-79) at risk worldwide in 2021 [1]. As the result of a change in lifestyle, characterized by reduced physical activity and increased obesity due to economic development and urbanization [2], the global number of individuals affected with diabetes may rise to 643 million by 2030 and 783 million by 2045. DR affects up to 80% of those who have had diabetes for 20 years or more, and at least 90% of new cases could have been reduced with proper treatment and monitoring of the eyes [3]. Early detection plays a pivotal role in clinical diagnosis, as retina degeneration is unidirectional and early treatment can decelerate further degradation.

A well trained learning-based model requires lots of labeled and/or annotated data for good generalization. However, large amounts of annotated data (e.g. lesion maps for the purpose of DR lesion segmentation) can be quite scarce, and obtaining clinical annotations is often a costly process. As deep learning progresses, especially in generative modeling, Generative Adversarial Networks (GANs) have proven to be capable of generating very high and very realistic images [4–7]. In the medical imaging field, GANs have shown to be a useful data augmentation tool for numerous downstream tasks [8–15].

### 1.1 Background

**Fig. 1.**
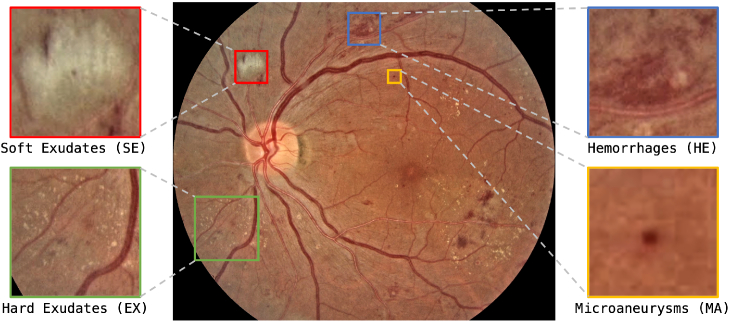
Common pathological lesions of DR in a retina fundus image. Image 0011_3.png from FGADR [17] Dataset.

The main cause of DR is micro-vascular changes in the retina that’s triggered by diabetes. Clinically, DR is split into five distinct classes according to International Clinical Diabetic Retinopathy (ICDR) [16] disease severity scale; no apparent retinopathy, {mild, moderate, severe} Non-Proliferative Diabetic Retinopathy (NPDR), and Proliferative Diabetic Retinopathy (PDR). NPDR, the more common type of DR, is a stage where the retina has visible signs of damage but not to the extent where new blood vessels are proliferating. PDR is a stage where the retina has become severely damaged, and blood vessels are now proliferating. These two stages may also be referred to as Early and Advanced DR respectively. Figure 1 shows the four most common pathological indicators of DR in a retina fundus image; Hard Exudates (EX), Soft Exudates (SE), Microaneurysms (MA) and Hemorrhages (HE).

In NPDR stage of DR, blood vessels of the retina are being blocked by sugar deposits and the walls of the blood vessels weaken under the increased pressure. Tiny bulges (MA) that protrudes from the cell walls can leak blood (HE) and fluid (EX or SE) into the retina and vitreous body (jelly-like substance that fills the center of the eye). Larger vessels start to dilate and become irregular in diameter. As more blood vessels become blocked, the disease progresses from mild to severe. This may also cause inflammation of the nerve fibers around the macula, a condition known as macular edema that requires treatment.

In PDR stage of DR, excess sugar in the blood stream will have blocked the tiny blood vessels at the back of the eye around the retina. This cuts off the blood supply that nourishes the area, leading to cell death. As a healing response by the body, leakages are closed off by the body’s clotting response, which triggers growth of new blood vessels in an attempt to restore blood flow back to the area. However, these blood vessels are often malformed, and are more prone to leak blood and fluids.

Prognosis of DR and/or segmentation of DR lesions is a time consuming and tedious task, thus it is desirable to expedite the process through automated means. The advantages of an automated solution brings consistency to the clinical workflow; higher throughput, and reduced human error due to factors such as being fatigued. Building prediction models for clinical diagnosis using fundus images – a common diagnostic technique based on photographs of the retina – is a challenging feat as the models require large amounts of annotated data. This is usually hard to obtain, especially with varying disease prevalence and class imbalances in existing public datasets.

Data augmentation [18] improves model generalization through enlargement of datasets. Commonly-used techniques include; rotating, flipping, scaling, cropping and color jittering. In recent years, generative models have developed vastly from being simple convolutional networks, *e.g.*, DC-GAN [19], to GANs that feature a variety of complex processes [5,7,20] to generate high-resolution and high-fidelity images. Thus, becoming a popular method of data augmentation for training models on small datasets that lacks diversity.

### 1.2 Related works

In medical imaging, generative models are mostly used to augment datasets that lacks annotations and/or labels. Shin et al. [8] uses Pix2Pix [21], an image translation networks, to generate synthetic multi-parametric MRI brain scans with tumors. An MRI-to-label network is first applied to T1-weighted brain scans to get brain segmentation maps. It is then combined with a tumor label that’s augmented with linear transforms (shift/enlarge/shrink) before using a label-to-MRI network to generate synthetic brain scans. Combining real data with synthetically generated data in the training cohort showed a small increase in segmentation Dice score. Frid-Adar et al. [9] used GANs to generate synthetic liver lesion ROIs, which subsequently is used to improve CNN classification of {Cyst, Metastasis, Hemangioma} lesions. They trained 3 separate DC-GAN models (one model for each lesion class), and also an AC-GAN [22] model (all 3 lesion classes combined). Their experiments showed that DC-GAN performs better than AC-GAN; achieving 85.7% sensitivity and 92.4% specificity, as compared to 78.6% and 88.4% respectively with standard CNN augmentations. Bhattacharya et al. [10] used a DC-GAN to generate synthetic Chest X-ray images to improve CNN pathology classification for {Infiltration, Atelectasis and No Findings} on the NIH ChestX-ray14 [23] dataset. By incorporating synthetic images with real images, classification accuracy increased from 60.3% to 65.3%. Sandfort et al. [11] trained a CycleGAN [24] to transform contrast CT scans into non-contrast scans, which is then used to augment segmentation models for {Kidney, Liver, Spleen}. Segmentation performance increased from 0.535 Dice to 0.747 Dice after incorporating synthetic scans into the training cohort.

Data augmentation can be especially useful for applications in DR, *e.g.*, applying to datasets such as DRIVE [25] or CHASE_DB1 [26] as they have a combined total of only 68 images with manually annotated segmentation labels. Zhou et al. [12] proposed DR-GAN, a multi-scale U-Net like architecture to synthesize high resolution fundus images using DR grade and lesion information. The generated images are then used for downstream tasks, *i.e.*, training DR grading and lesion segmentation models. Their method was evaluated on EyePACS [27], as well as their in-house FGADR [17] dataset, and have shown an increase in classification performance. Andreini et al. [13] split the synthesis task by proposing a two-stage pipeline for synthesizing high resolution fundus images. The first stage features a ProGAN [5] that is trained to generate semantic label maps of retina vessels. The generated label maps are then fed into the second stage, an image–to–image translation network [4], which generates realistic retinal images vasculature. The approach has been tested on DRIVE and CHASE_DB1 for retina vessel segmentation. Results showed that their model can obtain equal or better segmentation performance with respect to state-of-the-art techniques. Son et al. [14] also took a similar approach to synthesize high resolution retina images, however using lesions instead of vessels as the semantic label map. The first stage features a modified AC-GAN to generate class-conditioned semantic lesion labels at various DR grades for only Exudates. The generated lesion labels are subsequently fed into GauGAN [7], an image-to-image translation network, to synthesize high-resolution retina fundus images. Their method was evaluated on a lesion segmentation downstream task, and has shown to increase upon baseline methods.

In recent literature, there has also been several non GAN-based methods for augmenting datasets. Tan et al. introduced Foreign Patch Interpolation (FPI) [28] and Poisson Patch Interpolation (PPI) [29] frameworks to train networks for anomaly detection. FPI/PPI creates images with in-painted synthetic anomalies, along with the corresponding mask that identifies pixel alterations. Ghiasi et al. [30] proposed a Copy-Paste method to improve instance segmentation in natural images. Experiments showed they were able to train models that are up to 2x more data-efficient compared to standard augmentation on MS-COCO dataset. In the application of DR, Yu et al. [15] developed Multiple Lesions Insertion (MLI), which combines Copy-Paste with Poisson image blending to in-paint lesions directly onto healthy fundus images. Experiments show that by incorporating synthetic images with real images in the training cohort can increase CNN detection performance of DR, however, adding too much synthetic images can also hamper.

### 1.3 Contribution

Although several related works in literature aims to achieve similar goals, there are still shortcomings that can be addressed for a more diverse and robust model/pipeline. The combination of Copy-Paste and Poisson image blending as proposed by Yu et al. does indeed generated images are of high-resolution, however DR grade information of the synthetic images are ill-defined and thus the classification becomes a binary problem; referable or non-referable DR. In Andreini et al. and Son et al., the fundus images synthesis method was only applicable for one kind of mask, *i.e.*, vessels and Hard Exudates respectively.

In this paper we improve and extend the fundus image generation process, as proposed by Son et al., from generating fundus images with a single type of lesion (EX) to fundus images with the four most common lesions in literature [17,31] (MA, HE, EX, SE). This is achieved by a two-stage pipeline using Conditional StyleGAN in conjunction with GauGAN. The first stage we modify a StyleGAN [6] to be conditional in order to generate synthetic lesion maps of a specific DR grade. The second stage uses GauGAN to convert the lesion maps into fundus images.

We demonstrate that our two-staged pipeline is able to generate high-resolution photo-realistic retina fundus images of varying pathological severity through qualitative and quantitative evaluation. Experiments show our pipeline have beneficial performance in multiple subsequent downstream tasks; improving both DR grade classification and lesion segmentation.

Code and models of the pipeline can be accessed at [32]. We have also deployed a live demo, accessible at [33], to demonstrate the efficacy of the proposed pipeline.

## 2. Method

**Fig. 2.**
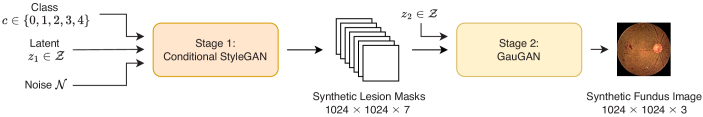
Proposed Two-Staged Retina Fundus Image Synthesis Pipeline

The task of synthesizing high resolution retina fundus images is split into two separate stages, as shown in Fig. 2. At the start of the pipeline, Conditional StyleGAN is used to synthesize lesion maps based on a specified DR grade (*i.e.*, grades 0 to 4). The second stage takes lesion maps and uses a GauGAN to generate synthetic retina fundus images. The lesions maps, used by GauGAN, can either be generated by Conditional StyleGAN or made manually through Copy-Paste method.

As the DR grade of a fundus image is determined by a set of rules outlined by the ICDR [16], the final grade takes into consideration of all present lesions in the image. Therefore, it is not possible to confer a DR grade for a lesion map made via Copy-Paste method, as the contribution weighting of each lesion is unknown. By using Conditional StyleGAN as the lesion map generator, where the trained model has learnt to approximate the rules, a DR grade can be associated with the synthetic lesion map. This further expands possible downstream tasks, e.g., dataset augmentation in DR grade classification settings.

### 2.1 Lesion mask generation

**Fig. 3.**
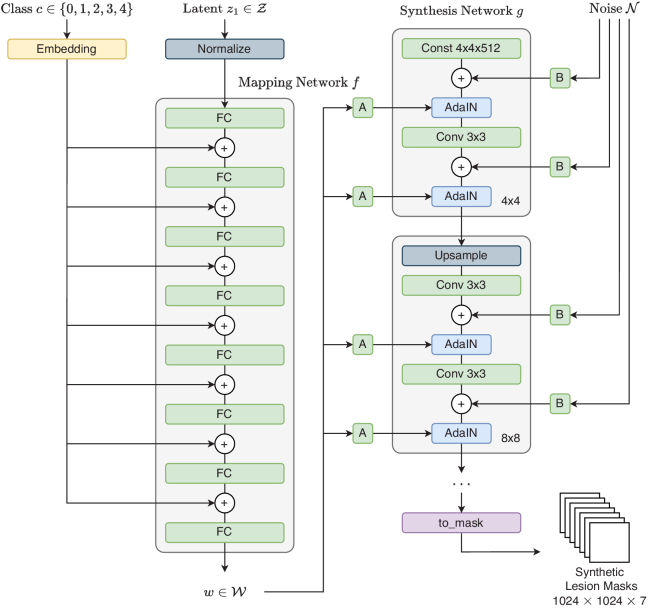
Architecture of Conditional StyleGAN Generator to generate synthetic lesion maps. Inputs to the network; the DR grade class c∈{0,1,2,3,4} , a latent "code" vector $z_{1}$ that’s sampled from a Gaussian distribution, and noise vector N that’s also sampled from a Gaussian distribution to provide stochasticity. The output of the network is a generated synthetic lesion map in one-hot encoding form.

To generate lesion masks of a specific DR grade, the generator of StyleGAN has been modified to also take DR grades as an input variable. Figure 3 shows the architecture of the Lesion Mask Generation network. The DR grade is passed through an embedding layer to get a representational vector of length 512. This is then added to the output of each linear layer in the mapping network, f . The mapping network maps a latent code $z_{1}\in Z$ and class c∈{0,1,2,3,4} to a style vector w∈W , such that; $w=f(\text{embedding}(c),z_{1})$ . Block ‘A’ is a learned affine transform layer, it takes in w and estimates its scale, $y_{s}$ , and bias, $y_{b}$ , parameters through 2 fully connected layers, such that; $y_{s}={\text{FC}}_{1}(w)$ , $y_{b}={\text{FC}}_{2}(w)$ and $\left\{{\text{FC}}_{1},{\text{FC}}_{2}\in A\right\}$ . These parameters are then subsequently used for the AdaIN [20] operation, defined by;

(1) $$\text{AdaIN}(x,y_{s},y_{b})=y_{s}\frac{x-\mu (x)}{\sigma (x)}+y_{b}$$

The purpose of AdaIn is to adjust the mean and variance of the content input, x , to match those of a particular style. This is achieved by first normalising the content, x , by subtracting its mean, μ(x) , and dividing by its standard deviation, σ(x) . The normalised content is then rescaled by multiplying $y_{s}$ and adding $y_{b}$ . To incorporate stochasticity, Gaussian noise, N , is broadcasted and added to all feature maps through a learned per-feature scaling Block ‘B’ (*i.e.*, a trainable weight parameter, with same cardinality as the noise vector, that is element-wise multiplied). The output of the network is a binary label with 7 channels. This forces the network to learn semantic lesion maps in one-hot encoding form instead of RGB class colors to avoid class ambiguity.

**Fig. 4.**
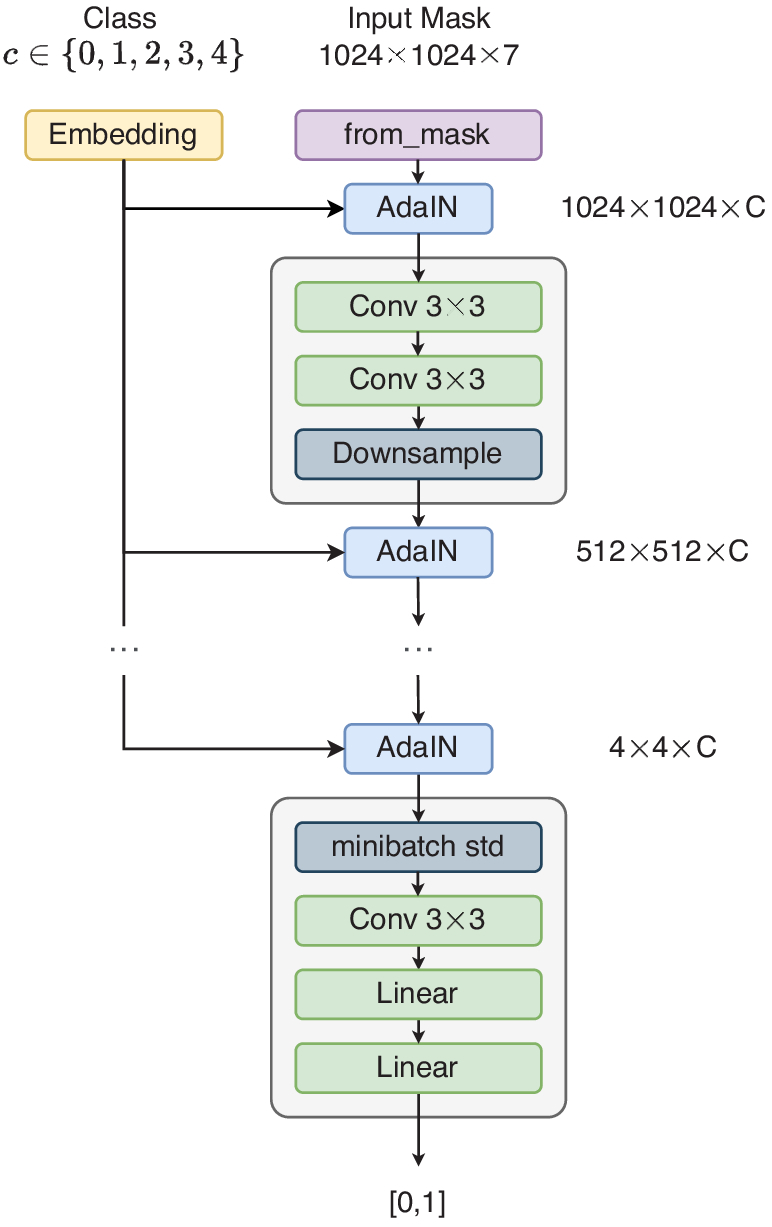
Architecture of Conditional StyleGAN Discriminator.

The discriminator for Conditional StyleGAN, shown in Fig. 4, is architecturally similar to the discriminator proposed in ProGAN [5]. However, it is also modified to take the embedding vector of the DR grade as context. The embedding vector is injected at the start of each resolution block using AdaIN. The weights for the embedding layer are only updated with the generator.

**Fig. 5.**
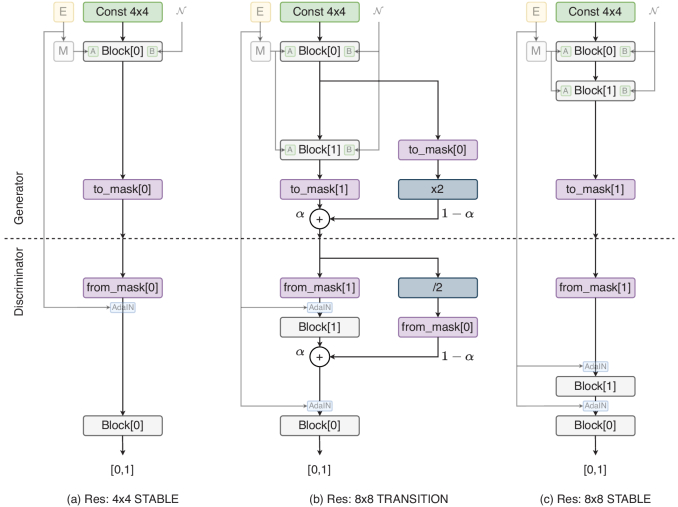
Conditional StyleGAN progressive growing of resolutions during training. Top half: Generator. Bottom half: Discriminator. (a) STABLE phase at 4 × 4 resolution, (b) TRANSITION phase at 8 × 8 resolution, (c) STABLE phase at 8 × 8 resolution. α parameter determines blending proportion between old block and new block.

Conditional StyleGAN is trained progressively, from a resolution of 4 × 4 to a desired resolution up to 1024 × 1024. The resolution doubles at each stage across two phases; TRANSITION and STABLE as shown in Fig. 5. In the TRANSITION phase, an alpha blending parameter that is proportional to the step count is used to blend the output of the upsampled image from the previous block with the output of the new resolution block. This is to ease the transition when the resolution is increased as the weights of the new block are randomly initialized.

The model is optimized using the Wasserstein GAN loss with Gradient Penalty (WGAN-GP) [34] for better stability during training, as defined in Eq. (2):

(2) $$\begin{array}{ll} L_{\text{D}}^{\text{WGANGP}} & =L_{\text{D}}^{\text{WGAN}}+\lambda E_{z∼p_{z},y∼p_{\text{data}}}[(||\nabla D{\left(\alpha y+(1-\alpha G(z))|{|}_{2}-1\right)}^{2}]\\ L_{\text{G}}^{\text{WGANGP}} & =-E_{z∼p_{z},y∼p_{\text{data}}}[D(G(z),y)] \end{array}$$

where G and D are the generator and discriminator networks respectively, z is a random latent Gaussian sample, y is a random image sample from the training dataset, α is the learning rate, λ is the gradient penalty coefficient and $L_{\text{D}}^{\text{WGAN}}$ is the original Wasserstein GAN loss.

### 2.2 Fundus image synthesis

**Fig. 6.**
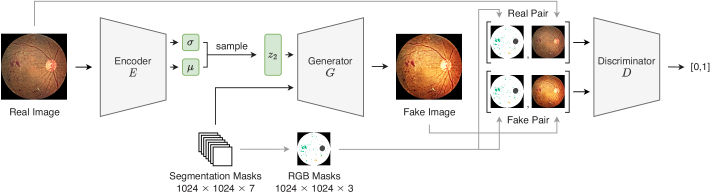
Complete GauGAN Architecture.

**Fig. 7.**
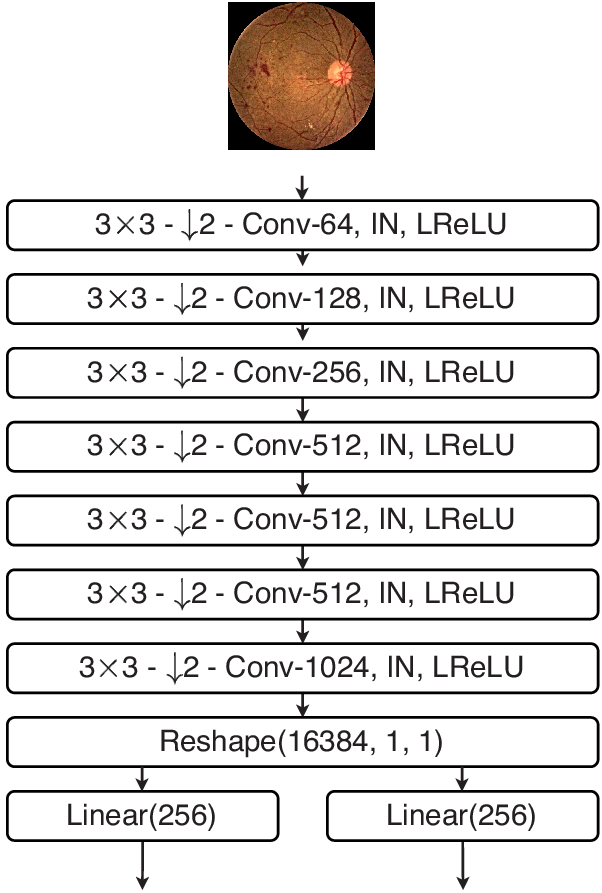
Encoder of GauGAN learns the Gaussian Distribution of the fundus images.

Lesions maps, real or synthetic, are passed through GauGAN to synthesise photo-realistic retina fundus images. Architecturally, GauGAN is can be regarded as a class-conditioned “VAE-GAN”, *i.e.*, a variational autoencoder that’s adversarially trained. Figure 6 shows the complete architecture of GauGAN during training. The variational formulation GauGAN, acts as a style guide for the Generator. The Encoder, Fig. 7, learns the mean and variance of a Gaussian Distribution using the fundus images as input.

**Fig. 8.**
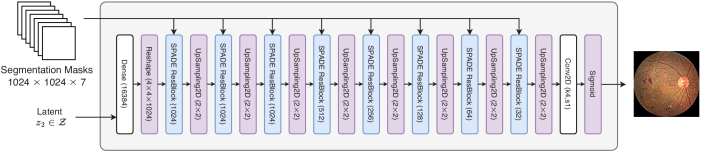
Generator of GauGAN converts generated lesion maps into fundus images.

The Generator, Fig. 8, follows a residual learning framework by design. It takes in latent vectors, $z_{2}\in Z$ , that are randomly sampled from a Gaussian distribution along with one-hot encoded semantic lesion maps to generate the retina fundus image. The variational formulation of sampling latent vectors from a Gaussian distribution introduces stochasticity, and allows the generator to achieve image diversity.

**Fig. 9.**
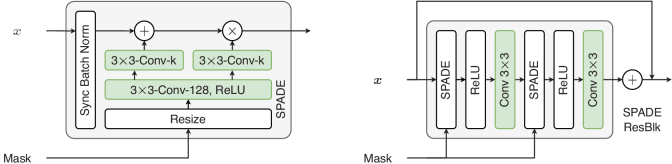
SPADE Block (L) and SPADE Residual Block (R)

To incorporate features from the mask, which is used as a conditioning factor, the authors proposed SPADE (SPatially-ADaptivE normalization), Fig. 9. SPADE is a normalization method that’s suitable for learning spatially adaptive affine parameters (scale and bias). This is done by learning individual sets of scaling and bias parameters for each semantic label map.

GauGAN is constrained with four loss functions to train the generator, and one loss function to train the discriminator. The loss functions for the generator starts with Expectation over the discriminator predictions, *i.e.*, standard GAN loss:

(3) $$L_{\text{GAN}}=-E_{z∼p_{z},y∼p_{data}}[D(G(z),y)]$$

The generator also uses a Feature Matching Loss that minimizes the discriminator predictions on generated images with original images, to align the feature space of the generator, such that;

(4) $$L_{\text{FM}}=||(D(y),D(G(z)))|{|}_{1}$$

Next, a VGG Feature Matching loss to ensure the generated images has similar visual perceptual quality to Imagenet. Where; VGG(x,m) is the feature map output of a pre-trained VGG-19 model, x is a feature input, and m∈M ={“relu1_1”, “relu2_1”, “relu3_1”, “relu4_1”, “relu5_1”} defines the VGG feature layers used:

(5) $$L_{\text{VGG}}=E_{z∼p_{z},y∼p_{data}}\sum_{i=1}^{5}\frac{1}{2^{i}}[||VGG(y,M_{i})-VGG(G(z),M_{i})|{|}_{1}]$$

Finally, KL-loss for the encoder so ensure the latent vectors are normally distributed:

(6) $$L_{\text{KLD}}=D_{\text{KL}}(q(z|x)||p(z))$$

The final loss of the Generator, $L_{\text{G}}$ , is the sum of the four defined losses above with equal weighting, shown in Eq. (7):

(7) $$L_{\text{G}}=L_{\text{GAN}}+L_{\text{FM}}+L_{\text{VGG}}+L_{\text{KLD}}$$

**Fig. 10.**
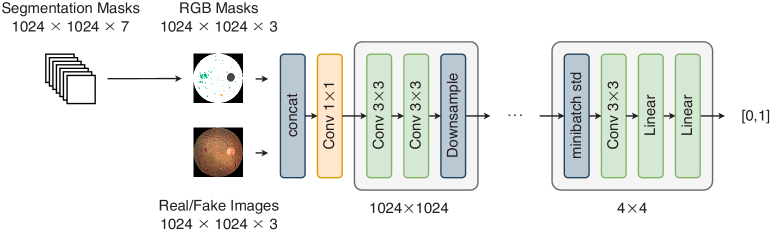
GauGAN Discriminator Architecture

Figure 10 shows the discriminator architecture of GauGAN. The discriminator takes in paired image-mask, in order to try and predict whether it’s real or generated. Instead of combining the real/fake RGB image with the mask in one-hot form, the one-hot masks are converted to RGB masks through an arbitrary colour class mapping. The discriminator is optimized using a Hinge loss only, shown in Eq. (8):

(8) $$\begin{array}{ll} L_{\text{D}}= & -E_{(x,y)∼p_{data}}[\min(0,-1+D(x,y))]\\ & -E_{z∼p_{z},y∼p_{data}}[\min(0,-1-D(G(z),y))] \end{array}$$

In all losses; G and D are the generator and discriminator networks respectively, z is a random latent Gaussian sample, and y is a random image sample from the training dataset. E is the Expectation operator and $D_{\text{KL}}$ is the Kullback–Leibler divergence.

GauGAN can synthesize high resolution fundus images from pathological lesion maps that are either; generated by Conditional StyleGAN or manually made via Copy-Paste method. Manually making hundreds of lesion maps for evaluation is far too time consuming, especially to ensure each lesion map is also medically accurate. To create a Copy-Paste lesion map for evaluation, 4 masks are randomly selected (one for each lesion; MA, HE, EX, SE) within a desired DR grade from the test set. The masks are then augmented and merged together. A free-hand paint tool can also be used to make synthetic lesion maps, however is reserved for future work.

### 2.3 Evaluation metrics

To evaluate the generative quality, we use Fréchet Inception Distance (FID), given by $d^{2}$ in Eq. (9). It is a metric commonly used to assess the quality of images created by generative models. The FID compares the distribution of generated images with the distribution of the ground truth images, and is defined by:

(9) $$d^{2}=||\mu_{1}-\mu_{2}|{|}^{2}+\text{Tr}(C_{1}+C_{2}-2\cdot \sqrt{C_{1}\cdot C_{2}})$$

where $\mu_{1}$ and $\mu_{2}$ is the feature-wise mean of the real and generated images, and $C_{1}$ and $C_{2}$ are the covariance matrix for the real and generated feature vectors, and Tr is the trace operation.

To measure the classification and segmentation performance for the downstream tasks, we use precision, recall, accuracy, and Dice/F1 score. Precision quantifies the number of positive predictions that actually belong to the positive class, whereas Recall quantifies the number of positive class predictions made out of all positive examples in the dataset. True positive TP is defined as correctly classified data, whereas true negative TN is defined as correctly rejected data. Similarly false positive FP denotes the incorrectly predicted data and false negative FN denotes the incorrectly rejected data.

(10) $$\text{Precision}=\frac{TP}{TP+FP}$$

(11) $$\text{Recall}=\frac{TP}{TP+FN}$$

(12) $$\text{Accuracy}=\frac{TP+TN}{TP+TN+FP+FN}$$

(13) $$\text{Dice / F1}=\frac{2\cdot \text{Precision}\cdot \text{Recall}}{\text{Precision}+\text{Recall}}$$

## 3. Experiment and results

### 3.1 Dataset

The Fine-Grained Annotated Diabetic Retinopathy [17], FGADR, dataset consists of 1,842 images and segmentation masks for six kinds of lesions. Each retina image is also graded with a DR grade score from 0 to 4 as defined by [16]. Most images were obtained from UAE hospitals and are the property of Inception Institute of Artificial Intelligence, Abu Dhabi, UAE. The DR grade distribution from Healthy (0) to PDR (4) are; 101, 212, 595, 647 and 287 respectively. Son et al. [14] visually inspected the dataset, and identified that one labeler has annotated the lesions in a very coarse manner. These annotations were very different compared to the annotations made by the other two annotators, and have shown to be problematic when training learning based models. Images with coarse segmentations were subsequently removed from the set, leaving a subset of 1,494 images (99, 157, 355, 605 and 278 images respectively for grades 0 to 4). For the experiments in this paper, the FGADR dataset is subsequently divided into training and testing set of 1,400 and 94 images respectively. A binary mask of seven distinct classes is then created for each fundus image; Background (BG), Vitreous Body (VB), Hard Exudates (EX), Hemorrhages (HE), Microaneurysms (MA), Soft Exudates (SE), and Optical Disk (OD). Annotations for the Optical Disk were not originally included in the dataset, the optical disks were manually segmented using makesense.ai.

### 3.2 Training protocol

The Conditional StyleGAN was trained progressively from a resolution of 4×4 to 256×256 , with batch sizes of (16, 16, 16, 16, 16, 8, 4) respectively. Each phase, TRANSITION and STABLE, was trained with 10,000 steps using the Adam optimizer with a learning rate of 2e-3 for both generator and discriminator networks. The optimizer’s parameters were reset at the start of each phase. Training took 28hrs in total. GauGAN was trained to a resolution of 1024 × 1024. The network was trained with a batch size of 4 for 100 epochs. The generator and discriminator were trained using the Adam optimizer with a learning rate of 1e-4 and 4e-4 respectively. Training took approximately 10.5hrs in total. Both models were trained on a machine equipped with an Intel i7-6700K CPU and Nvidia Titan Xp GPU using Tensorflow 2.0 + Keras. To connect the pipeline between Conditional StyleGAN and GauGAN, the generated lesion maps by Conditional StyleGAN were upsampled by 4x via Nearest Neighbor Interpolation.

### 3.3 Synthetic perceptual quality

The perceptual quality of images generated by GauGAN was evaluated against the test set of images from FGADR using FID. This gives a quantitative measure to evaluate how close the generated images are w.r.t. the real images in terms of image features. GauGAN was tasked with synthesising retina fundus images from synthetic masks made via Copy-Paste, as well as synthetic masks that’s generated by Conditional StyleGAN. Table 1 shows the results:

**Table 1.** Fréchet Inception Distance of Synthetic vs Real Images

	FID Score
Copy-Paste Generation	33.410
Conditional StyleGAN Generation	39.056

**Fig. 11.**
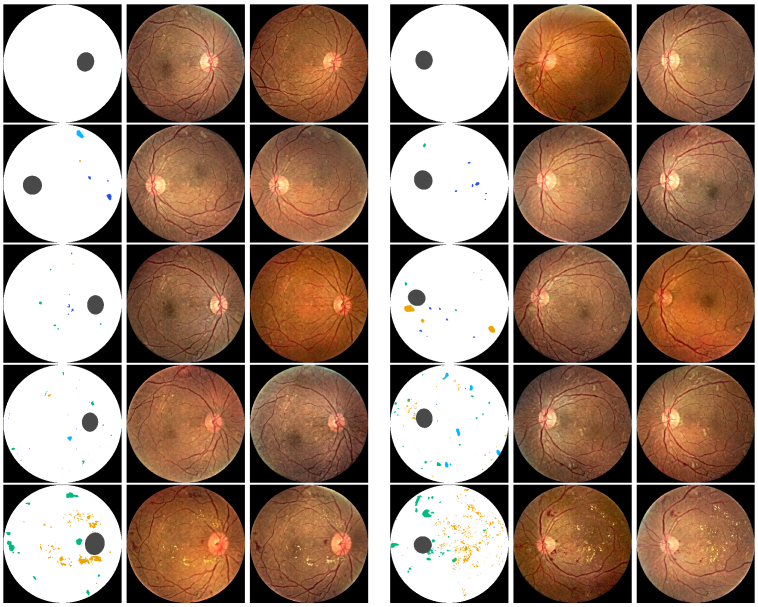
Example retina fundus images synthesized from Copy-Paste lesion maps. Rows top to bottom: approximate DR grades 0 to 4. Columns 1,4: artificial hand crafted lesion maps made via Copy-Paste method shown in RGB representation. Columns 2,3,5,6: example synthetic fundus images generated by GauGAN.

Figure 11 shows example images generated by GauGAN from semantic lesion maps made via Copy-Paste method. To create a mask via Copy-Paste method, lesion masks were selected from random subjects of a specific DR grade. One mask for each kind of DR lesion. The four lesion masks, along with the optical disk (OD), were painted on a white circular disc denoting the vitreous body (VB) with a black background (BG). To ensure the class of each pixel is mutually exclusive, *i.e.*, the aggregation result is one-hot, the class priority is selected by the order of: BG → OD → EX → HE → SE → MA → VB. 20 masks were randomly made from each of the 5 DR grades (100 masks total).

**Fig. 12.**
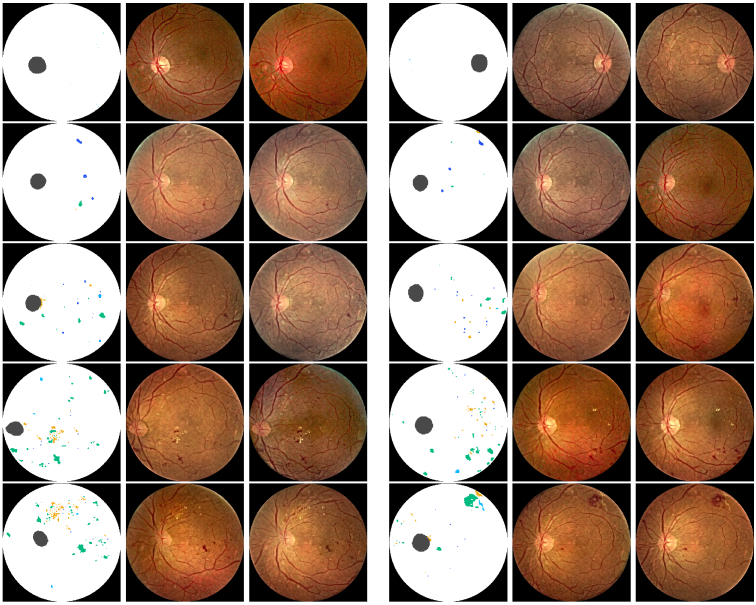
Example retina fundus images synthesized from Conditional StyleGAN generated lesion maps. Rows top to bottom: DR grades 0 to 4. Columns 1,4: synthetically generated lesion maps based on DR grade by Conditional StyleGAN shown in RGB representation. Columns 2,3,5,6: example synthetic fundus images generated by GauGAN.

Figure 12 shows retina fundus images generated using the full proposed pipeline. Conditional StyleGAN was used to generate several lesion maps of each DR grade. The generated masks were upscaled by a factor of four with nearest neighbor interpolation, and subsequently fed into GauGAN to synthesize the fundus image. 20 images were generated for each DR grade, which were then used to calculate the FID score w.r.t. the test set. N.B. masks generated by Conditional StyleGAN and images generated by GauGAN were **not cherry picked** for the calculation.

Achieving a FID score of 30-40 is inline with performance reported for state-of-the-art GANs [35]. GANs are notoriously unstable in their training process, and are known to hallucinate undesirable structures [36] if the harmonic balance between generator and discriminator is not met. We are confident in the stability and robustness of our model that the synthesized images are medically plausible.

### 3.4 Dataset Balancing

In this experiment, the method of GAN sampling for dataset balancing is evaluated against classical methods, namely; undersampling and oversampling. Table 2 shows the original baseline distribution of classes in the training set. Whilst it is not the most unbalanced DR dataset that’s publically available, the majority class (grade 3) still out numbers the minority class (grade 0) by 6:1. In undersampling method, images are randomly discarded so that the sample count of each class matches the least representative class. Conversely, in oversampling method, images of each class are repeatedly randomly sampled from their respective class to match the count of the most representative class. For GAN sampling, additional images are generated for each respective class to match the the count of the majority class. An additional 25 images have also been generated to ensure all classes contain at least some synthetic examples.

**Table 2.** Class distribution of training dataset

DR_grade	Baseline	Undersampling	Oversampling	GAN Sampling
0	96	96	575	600 (+504)
1	145	96	575	600 (+455)
2	330	96	575	600 (+270)
3	575	96	575	600 (+25)
4	254	96	575	600 (+346)

Total	1400	480	2875	3000

An InceptionResNetV2 was trained from scratch for each class. Each network is trained using the Adam optimizer with a batch size of four and learning rate of 1e-4 for approximately 50 epochs. Standard Binary-Crossentropy is used as the loss function. Data augmentation include random $360^{\circ }$ rotation with left-right and top-down random flipping. Table 3 shows the results.

**Table 3.** Accuracy, Precision, Recall and F1 scores of dataset balancing methods. Best attained value is highlighted in bold.

Method	Accuracy	Precision	Recall	F1
Baseline	0.702	0.690	0.702	0.676
Undersample	0.574	0.608	0.574	0.530
Oversample	0.660	0.678	0.660	0.656
GAN Sampling	**0.713**	**0.762**	**0.713**	**0.708**

**Fig. 13.**
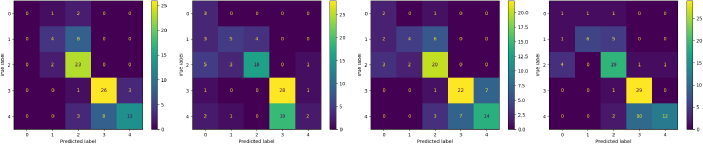
Confusion Matrix of DR grade classification. L-to-R: Baseline, Undersampling, Oversampling, GAN Sampling.

Experiments show undersampling and oversampling methods, achieving an accuracy of 0.57 and 0.66 respectively, underperforms as compared to baseline and GAN Sampling, which achieved 0.70 and 0.71 accuracy respectively. To a certain extent, this is quite anticipated. Undersampling severely hampers the dataset diversity as it discards potentially useful data, whilst oversampling induces overfitting of minority classes. This is reflected by the Confusion Matrix in Fig. 13, which shows the performance of InceptionResNetV2 on predicting DR grades. InceptionResNetV2 performed terribly on predicting grade 4 only in the Undersampling case. This affirms the notion that useful image features, which best represent the class, has been discarded through random selection. GAN Sampling resulted the best performing network in all metrics. Whilst there is only a small gain in accuracy compared to baseline (0.71 as to 0.70), it achieved a much higher precision (0.76 compared to 0.69), indicating fewer false positives. The small gain in accuracy is likely attributed to the fact that data augmentation (random rotation and flipping in this setting) is already a strong method of increasing data diversity.

## 4. Downstream tasks

The synthesis pipeline is evaluated for its effectiveness in improving DR grade classification and also lesion segmentation downstream tasks. The InceptionResNetV2 was chosen as an out-of-box baseline network for DR grade classification, whilst a vanilla U-Net was chosen as the baseline network for lesion segmentation. Both tasks were trained under three scenarios: only on synthetic data (Fake), only on real data (Real), and pre-training on synthetic data with fine-tuning on real data (Fine). For testing, the trained networks were evaluated on real images, *i.e.*, the test set images from FGADR only. Each scenario is trained using the Adam optimizer with a batch size of four and learning rate of 1e-4 for approximately 50 epochs. Data augmentation include random $360^{\circ }$ rotation with left-right and top-down random flipping.

### 4.1 Classification

Table 4 shows the performance of InceptionResNetV2 on DR grade classification. Training with only synthetic data and testing on real data performed the worst. This is expected as GAN models are trained to capture the overall distribution of the dataset, and likely to have missed out some fine grain features. Despite this, pre-training InceptionResNetV2 with synthetic data followed by fine tuning on real data showed noticeable improvement in classification performance. Following with a Wilcoxon signed-rank test between the predicted classes of Real and Fine scenarios resulted in a p-value of <0.05 , highlighting significant difference.

**Table 4.** Accuracy, Precision, Recall and F1 scores for InceptionResNetV2 grading DR of real retina fundus images. Best attained value is highlighted in bold, with (⋅) denoting Confidence Interval at 95%. Wilcoxon signed-rank test between Real and Fine yielded a p-value of 0.025 < 0.05

	Accuracy	Precision	Recall	F1
Fake	0.431 (0.042)	0.375 (0.028)	0.402 (0.052)	0.353 (0.039)
Real	0.628 (0.041)	0.548 (0.086)	0.472 (0.034)	0.449 (0.028)
Fine	**0.646 (0.010)**	**0.566 (0.045)**	**0.573 (0.066)**	**0.547 (0.044)**

**Fig. 14.**
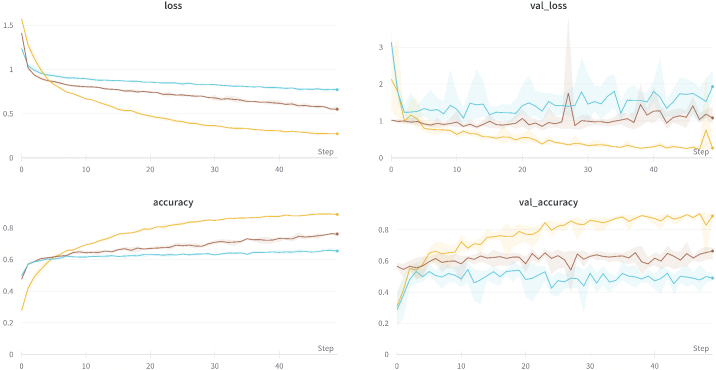
Training loss, validation loss, training accuracy and validation accuracy for Fake (Yellow), Real (Blue) and Fine (Brown) scenarios.

Figure 14 shows graphs for training loss, validation loss, training accuracy and validation accuracy respectively. It can be seen that the fine tuning run has attained a much lower loss and higher accuracy as compared to training on the real data directly. As the experiment is run in a 4-fold cross validation setting, it can be seen that the uncertainty of each respective run do not overlap. This highlights significant difference as reflected by the Wilcoxon signed-rank test.

### 4.2 Segmentation

Table 5 shows the performance of U-Net on DR lesion segmentation. Like with the classification downstream task, there were noticeable, albeit small, improvements in performance when the network is first pre-trained with synthetic data.

**Table 5.** Dice/F1 score for each lesion and optical disc segmentation. Best attained value is highlighted in bold.

	EX	HE	SE	MA	OD
Fake	0.283	0.325	0.269	0.132	0.916
Real	0.510	0.518	0.657	**0.299**	0.955
Fine	**0.533**	**0.552**	**0.693**	0.274	**0.959**

**Fig. 15.**
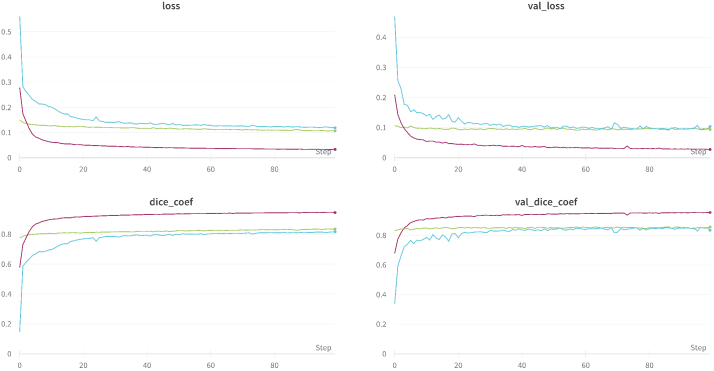
Segmentation training loss, validation loss, training dice and validation dice for Fake (Red), Real (Blue) and Fine (Green) scenarios.

Segmentation performance of the model is reflected by the training loss, validation loss, training dice and validation dice graphs as shown in Fig. 15. The networks loss for both training and validation plateaus much faster compared to starting from random initialization, and are able to achieve much lower values in the same amount of epochs. Even though the performance gain was small, using pretrained weights does not require many epochs for fine tuning, which saves on computation time.

## 5. Discussion

High resolution images are key for accurate diagnosis. As DR lesions are usually very small, and only occupy a few pixels, a full resolution fundus image can be as big as 1024 × 1024px. Whilst GANs in literature, such as StyleGAN and Pix2PixHD, are able to synthesize images of very high resolutions, it can also be computationally expensive and GPU memory intensive to train these models. Downscaling images can circumvent this, however, smaller lesions may disappear. This is especially the case for MA, as seen by the low Dice score in Table 5.

Synthesis stability was achieved at a resolution of 256 × 256 for Conditional StyleGAN and 1024 × 1024 for GauGAN. Higher resolutions caused the networks to become unstable during training where images generated contained artifacts that, just through visual inspection, was indicative that it is not realistic or biologically plausible, *e.g.*, checkerboard patterns for lesions. Furthermore, as the resolution increases exponentially, the training time increased from a few hours to a few days. Adequate quality can be achieved but requires further fine tuning of hyper-parameters, which can be a computationally expensive task. Lesion masks generated by Conditional StyleGAN were upsampled via nearest-neighbor, which should have minimal impact from upsampling artifacts.

There are far too many GAN architectures available in literature and is time costly to test every single one. However, in recent literature, new generative models, *e.g.* diffusion models, have been proposed that can also generate high resolution and high fidelity images with competitive performance. The downside to these models is a trade-off decision between model complexity, pace of experimentation, image quality, and diminishing rate of return.

The Cut-Paste method of generating synthetic masks in this work is currently quite simple. Lesion masks are used in it’s entirety based on the DR grade. For future work, we would definitely like to introduce a bit more variability, *i.e.*, enabling linear transformations, such as; translate, rotate, enlarge and shrink lesions similar Shin et al. [8], to create more rich and diverse examples.

Additionally, as our tool has potential interactive component, we would like to build an interactive paint tool for synthesising lesions maps in future work. This interactivity can allow more flexibility as compared to manipulating lesions masks by code. Improving Cut-Paste method can mitigate the need of a difficult-to-train GAN model, however, as discussed earlier that the final DR grade is governed by the contribution of all lesions present. This could be solved by introducing a model that can classify DR grades from lesion maps instead of DR images.

Furthermore, as part of future work, it would be incredibly beneficial to have feedback from trained opthamologists on the quality of synthetic images. This would give a true gold standard quantitative evaluation of synthetically generated images in place of FID. We would like to see our proposed pipeline to be deployed as part of a real clinical workflow, which for example, another possible use case is to train new opthamologists.

## 6. Conclusion

In this paper, we introduced a pipeline for generating high-fidelity retina fundus images by splitting the task into two distinct components; a Conditional StyleGAN to synthesize semantic lesion maps based on a specified DR grade, and GauGAN to synthesize high resolution fundus images from the semantic lesion maps. GauGAN was able to synthesize images from lesion maps that were both hand-crafted via a Copy-Paste method, as well as from lesion maps that’s generated by Conditional StyleGAN. The perceptual quality of the images generated were qualitatively acceptable, and quantitatively achieved a FID score of 33.410 and 39.056 respectively. This is within expected range of well-performing GANs in literature [35,37]. The pipeline was further evaluated for its use in a downstream task of boosting DR grade classification and lesion segmentation performance. Both cases showed an increase in performance, improving scores in all relevant metrics.

## Data Availability

Data underlying the results presented in this paper are available in [17]. Code underlying the results presented in this paper are available in [32].
